# Supplementary material for: Development and Application of Healthiness Indicators for Commercial Establishments That Sell Foods for Immediate Consumption
Source: Foods. 2021 Jun 21;10(6):1434. doi: 10.3390/foods10061434 (PMC8235135; doi:10.3390/foods10061434)
Supplement: Supplementary file 1 [file foods-10-01434-s001.zip › foods-1244288-supplementary.pdf]

UNIVERSITY FOOD ENVIRONMENT EVALUATION QUESTIONNAIRE

University: __________________ **(ID:** __________**)**
Assessor: ______________________ **(ID:** __________**)**
Establishment: _______________ **(ID:** __________**)**
________________________**(ID Quest.**__________ **)**
Date: ____ **/**____ **/**______
Start time: ___ ___**:** ____ ____
End time: ___ ___**:** ____ ___

CHARACTERIZATION OF ESTABLISHMENT

1. TYPE OF ESTABLISHMENT:
(1) Buffet restaurant by weight
(2) À la carte/fixed dish restaurants
(3) All-you-can-eat buffet restaurants, fixed price
(4) Snack bar
(5) Bar
(6) Cafeteria
(7) Candy store
(8) Mixed (snack bars or cafeterias and buffet by weight or la carte/fixed dish restaurants)

2. LOCATION OF ESTABLISHMENT
(1) Within the university (2) In surrounding area

3. THE ESTABLISHMENT OFFERS (you *may choose more than one option*):
(1) Snacks (2) Meals (3) items from candy store

3.1 In the case of MEALS (you *may choose more than one option*):

(1) A la carte	(5) Portioned Buffet
(2) Meal by weight	(6) Daily special/Full meal
(3) Free Buffet	(7) Eat-as-much-as you-can
(4) Mixed Buffet	(8) Other (____________________)

4. OPENING HOUR of the establishment:
4.1. Monday to Friday (0) No (1) Yes
4.1.1 Opens ____ 4.1.2 Closes ____

4.2. Saturday (0) No (1) Yes
4.2.1 Opens ____ 4.2.2 Closes ____

4.3. Sunday (0) No (1) Yes
4.3.1 Opens ____ 4.3.2 Closes ____

4.4. Holidays (0) No (1) Yes
4.4.1 Opens ____ 4.4.2 Closes ____

4.5 Other: _________________________
4.5.1 Opens ____ 4.5.2 Closes ____

5. What are the BUSIEST TIME(S) during opening hours between Monday and Friday:
5.1________: _______to ________: _________
5.2 ________: _______to ________: _________

6. Does the place offer alternative FORMS OF PAYMENT (*you may choose more than one option*):

(1) Cash	(4) Meal voucher
(2) Debit card	(5) Other
(3) Credit card	(___________________)

NOTES ON THE ENVIRONMENT

7. DOES THE ESTABLISHMENT HAVE:

7.1 Micro-wave available for use by customers?	(0) No	(1) Yes
7.2 Structure (table, counter etc.) for eating in?	(0) No	(1) Yes
7.3 Freely accessible shelves displaying items such as cookies, desserts, candy, cereal bars?	(0) No	(1) Yes
7.4 Freely accessible display refrigerators (glass door/ with stickers) for drinks?	(0) No	(1) Yes
7.5 Freely accessible display freezers (glass door/ with stickers) for ice creams and ice lollies?	(0) No	(1) Yes
7.6 Freely accessible counter for desserts and sweets	(0) No	(1) Yes
7.7 Freely accessible counter for drinks	(0) No	(1) Yes
7.8 Snacks displayed next to cash desk	(0) No	(1) Yes
7.9 Water fountain with potable water	(0) No	(1) Yes
7.10 Free filtered water	(0) No	(1) Yes

In the case of restaurant serving "meals by weight", free buffet, mixed buffet, portioned buffet or other type of service with display of preparations, respond to questions 8 and 9. In the case of other establishments, mark "Does not apply" to questions 8 and 9 and continue to complete question 10:

8. What is displayed AT THE START OF THE BUFFET FLOW (*you may choose more than one option*):

(1) Salads	(5) Meats/barbeque
(2) Fruits	(6) Accompaniments
(3) Drinks	(7) N/A
(4) Desserts	(8) Other food/preparation: (_______________________)

9. What is displayed AT THE START OF THE BUFFET FLOW (*you may choose more than one option*):

(1) Salads
(2) Fruits
(3) Drinks
(4) Desserts
(5) Meats/barbeque
(6) Accompaniments
(7) N/A
(8) Other food/preparation
(____________________)

10. If there are freely accessible shelves displaying food items, WHAT PRODUCTS ARE AVAILABLE (*you may choose more than one option*)?

(1) Cookies sweets with filling
(2) Cookies sweets without filling
(3) Packets of savory snacks or savory cookies without filling (e.g. Clube Social, drink)
(4) Wholemeal cookies
(5) Cereal bars
(6) Candies, sweets, chocolates
(7) Other sweets (cakes, honey bread, sweetened breakfast cereal)
(8) N/A
(9) Other food/preparation
(____________________)

11. If there are display refrigerators (glass door/ with stickers) or freely accessible shelves for drinks, WHAT PRODUCTS ARE AVAILABLE (*you may choose more than one option*)?

(1) Natural fruit juice (100% Juice)
(2) Fruit juice or fruit nectar based drinks
(3) Flavored water (e.g. Aquarius Fresh, Natural Plus and Neutra)
(4) Coconut water
(5) Flavored milk or milk-based beverages or industrialized yogurt
(6) Soy-based drinks
(7) Tea or mate
(8) Energy drinks (e.g. Red Bull)
(9) Sports drinks (e.g. Gatorade)
(10) Guarana drink
(11) Sodas (including flavored water)
(12) N/A
(13) Other food/preparation
(____________________)

INFORMATION

12. Which of the following items are AVAILABLE TO CUSTOMERS:

12.1 Menu: (0) No (1) Yes
12.2 Price: (0) No (1) Yes
12.3 Nutritional information: (0) No (1) Yes
12.3.1 Energetic value: (0) No (1) Yes
12.3.2 Allergens: (0) No (1) Yes
12.3.3 Macronutrients: (0) No (1) Yes
12.3.4 Micronutrients: (0) No (1) Yes

12.4 IF YES, for the corresponding item, indicate (*you may choose more than one option*):

12.4.1 MENU:
(1) Individual
(2) Table display
(3) Banner/totem/TV
(4) Internet
(5) Individual menu outside
(6) Other (________________)

12.4.2 PRICE:
(1) Individual
(2) Table display
(3) Banner/totem/TV
(4) Internet
(5) Individual menu outside
(6) Other (________________)

12.4.3 NUTRITIONAL INFORMATION
(1) Individual
(2) Table display
(3) Banner/totem/TV
(4) Internet
(5) Individual menu outside
(6) Other

DRINKS, FOODS AND PREPARATIONS

13. ARE GREENS AND/OR LEGUMES OFFERED (Do not consider roots and tubercules) (*you may choose more than one option*):

13.1 RAW?
(0) No (*go to q13.2*) (1) Yes

13.1.1 AS COMPOSITE PREPARATIONS ONLY WITH GREENS / LEGUMES?
(0) No (1) Yes

13.1.2 AS PART OF PREPARATIONS?
(0) No (1) Yes

13.2 COOKED (including soups)?
(0) No (*go to q14*) (1) Yes

13.2.1 AS COMPOSITE PREPARATIONS ONLY WITH GREENS / LEGUMES?
(0) No (1) Yes

13.2.2 AS PART OF MIXED PREPARATIONS:
(0) No (1) Yes

13.2.3 AS PART OF PREPARATIONS WITH SAUCE (e.g. white sauce):
(0) No (1) Yes

14. IS THERE WHOLE GRAIN RICE?
(0) No (1) Yes

15. ARE THERE LEGUMES?
(0) No (1) Yes

16 . ARE THERE CONDIMENTS AND SAUCES:
(0) No (*go to q17*) (1) Yes

16.1 Salt? (0) No (1) Yes

16.2 Oil? (0) No (1) Yes

16.3 Olive oil? (0) No (1) Yes

16.4 Readymade dressings (including salad dressing, ketchup, mayonnaise and mustard)?
(0) No (1) Yes

16.5 Homemade dressings?
(0) No (1) Yes

16.6 Soy sauce?
(0) No (1) Yes

16.7 Other sauces?
(0) No (1) Yes (_______________)

17. ARE THERE SANDWICHES OR CREPES?
(0) No (1) Yes

18. ARE THERE FRIED/BAKED SAVORY SNACKS?
(0) No (1) Yes

19. ARE THERE FRESH FRUITS OR FRUIT SALADS?
(0) No
(1) Yes, throughout the day
(2) Yes, only during main meals

20. IS THERE WATER FOR SALE?
(0) No (1) Yes

21. ARE THERE NATURAL FRUIT JUICES OR PREPARED WITH FROZEN PULP (*you may choose more than one option*)?
(0) No (1) Yes, unsweetened (2) Yes, ready sweetened

22. ARE THERE INDUSTRIALIZED JUICES IN COOLERS/BOTTLES (*you may choose more than one option*)?
(0) No (1) Yes, unsweetened (2) Yes, ready sweetened

23. IS THERE SUGAR ON THE TABLES?
(0) No (1) Yes

24. IS THERE SWEETENER?
(0) No (1) Yes

CONVENIENCE ITEMS
(INCLUDING CANDY STORE)

**25.** ARE THERE:			*NO. OF DIFFERENT TYPES*
25.1 Candy and chocolate bars	(0) No	(1) Yes	________
25.2 Candy (including chewing gum)	(0) No	(1) Yes	________
25.3 Cereal bars	(0) No	(1) Yes	________
25.4 Breakfast cereal	(0) No	(1) Yes	________
25.5 Cream-filled cookies	(0) No	(1) Yes	________
25.6 Cookies without filling	(0) No	(1) Yes	________
25.7 Packaged savory snacks or crackers without filling	(0) No	(1) Yes	________
25.8 Whole grain cookies	(0) No	(1) Yes	________
25.9 Other sweet (honey bread, cake, muffin,)	(0) No	(1) Yes	________
25.10 Flavored water (e.g. Aquarius Fresh, Natural Plus and Neutra)	(0) No	(1) Yes	________
25.11 Coconut water	(0) No	(1) Yes	________
25.12 Soda (including H20)	(0) No	(1) Yes	________
25.13 Fruit juice or fruit nectar based drinks	(0) No	(1) Yes	________
25.14 Ready-to-drink tea (e.g. Ice Tea, mate)	(0) No	(1) Yes	________
25.15 Sports drinks (Gatorade)	(0) No	(1) Yes	________
25.16 Energetic (e.g. Red Bull)	(0) No	(1) Yes	________
25.17 Guarana drink	(0) No	(1) Yes	________
25.18 Soy-based industrialized beverage	(0) No	(1) Yes	________
25.19 Flavored milk or milk-based beverages or industrialized yogurt	(0) No	(1) Yes	________
25.20 Alcoholic drinks	(0) No	(1) Yes	________
25.21 Coffee (self-service)	(0) No	(1) Yes	________
25.22 Others	(0) No	(1) Yes	________

(_____________________________________)

26. What is the cost of the **SMALLEST** PORTION OR CHEAPEST ITEM AVAILABLE AND SOLD SEPARATELY for the following foods:

26.1 Meal
26.1.1 Daily special: R$ _____________ (0) N/A
26.1.2 By weight: R$ _____________ (0) N/A

26.2 French fries
26.2.1 Portion: R$ ____________ (0) N/A
26.2.2 By weight: R$ ____________ (0) N/A

26.3 Fruit or fruit salad *(which ever is cheaper)*
26.3.1 Portion R$ ____________ (0) N/A
26.3.2 By weight: R$ ____________ (0) N/A

26.4 Cheapest sweet or desert
(Do not consider candy store item)
26.4.1 Portion R$ ____________ (0) N/A
26.4.2 By weight: R$ ____________ (0) N/A

26.5 Sandwich R$ ____________ (0) N/A

26.6 Fried savory snack R$ ____________ (0) N/A

26.7 Baked savory snack R$ ____________ (0) N/A

26.8 Cheapest sweet snack
(Do not consider candy store item)
26.8.1 Price: R$ ____________ (0) N/A
26.8.2 Weight:____________ (0) N/A

26.9 Cereal bar
26.9.1 Price: R$ ____________ (0) N/A
26.9.2 Weight: ____________ (0) N/A

26.10 Filled sweet cookie
26.10.1 Price: R$ ____________ (0) N/A
26.10.2 Weight: ____________ (0) N/A

26.11 Unfilled sweet cookie
26.11.1 Price: R$ ____________ (0) N/A
26.11.2 Weight: ____________ (0) N/A

26.12 Packets of savory snacks or unfilled savory cookies
26.12.1 Price: R$ ____________ (0) N/A
26.12.2 Weight: ____________ (0) N/A

26.13. Whole grain cookies
26.13.1 Price: R$ ____________ (0) N/A
26.13.2 Weight: ____________ (0) N/A

27. What is the cheapest item AVAILABLE AND SOLD SEPARATELY OF THE FOLLOWING DRINKS:

27.1 Soda
Volume: ______ml
R$ ____________________ (0) N/A

PRICES AND PROMOTIONS

27.2 Juice/natural guarana / ice tea/ mate
Volume: ____ml
R$ ____________________ (0) N/A

27.3 Natural or pulp fruit juice (300 ml)
R$ ____________________ (0) N/A

27.4 Industrialized fruit juice (100%) (300 ml)
R$ ____________________ (0) N/A

27.5 Nectar (e.g. Sufresh, Del Vale, Da Fruta) (350 ml)
R$ ____________________ (0) N/A

27.6 Juice based drinks (200 ml) (Kapo)
R$ ____________________ (0) N/A

27.7 Mineral water
Volume: ______ml
R$ ____________________ (0) N/A

27.8 Sport drink (Isotonic/replacement drinks) (500ml)
R$ ____________________ (0) N/A

27.9 Energy drinks (200-300ml)
R$ ____________________ (0) N/A

27.10 Soy based drinks (200ml)
R$ ____________________ (0) N/A

27.11 Milk based or derivate drinks (includes yoghurt and fermented milk)
Volume: ___ml
R$ ____________________ (0) N/A

27.12 Mixed milk and fruit drinks
Volume: __________ml
R$ ____________________ (0) N/A

27.13 Alcoholic drinks (350ml)
R$ ____________________ (0) N/A

28. Is there the option of asking for LARGER PORTIONS OF FOODS AND DRINKS OFFERED? (e.g. choose 500 ml soda instead of 350ml or have 200g more fries)
0) No (1) Yes

28.1 IF YES, theses prices are proportionally:
(1) Lower (2) the same (3) Higher

29. Is there the option of ASKING FOR ½ PORTION OR REDUCED PORTIONS?
(0) No (1) Yes (2) N/A

29.1 IF YES, theses prices are proportionally:
(1) Lower (2) the same (3) Higher

30. Does the place OFFER COMBO/PROMOTION OPTION WITH END PRICE LOWER THAN THE SUM OF PRICES OF EACH COMPONENT OF THE COMBINATION?
(0) No (1) Yes (2) N/A

31. Is there the option of SUBSTITUTING FRIES FROM THE SPECIAL/READY/COMBOS/PROMOTIONS WITH SALAD OR GREENS/ COOKED LEGUMES **AT NO EXTRA COST**?
(0) No (1) Yes (2) N/A

32. Does the establishment offer the option of SUBSTITUTING RICE FROM THE SPECIAL/READY/COMBOS/PROMOTIONS WITH WHOLE GRAIN RICE **AT NO EXTRA COST**?
(0) No (1) Yes (2) N/A

33. Does the establishment offer the option of SUBSTITUTING WHITE BREAD FROM THE SPECIAL/READY/COMBOS/PROMOTIONS WITH WHOLE GRAIN BREAD **AT NO EXTRA COST**?
(0) No (1) Yes (2) N/A

34. Does the establishment offer the option of SUBSTITUTING THE SODA FROM THE COMBOS/PROMOTIONS WITH FRESH OR PREPARED NATURAL JUICES WITH FROZEN PULP OR WITH WATER, **AT NO EXTRA COST**?
(0) No (1) Yes (2) N/A

35. Does the place offer PROMOTIONAL/SUBSIDIZED PRICES FOR UNIVERSITY STUDENTS?
(0) No (1) Yes

ADVERTISING

NB. Use the table below to fill in the forms of presentation of advertising

Form of presentation
(1) banner/supplier sign<br>(2) banner/sign of establishment<br>(3) uniform<br>(4) replica of product<br>(5) menu<br>(6) on the product

36. Is there any advertising in the establishment for FRESH FRUIT / FRUIT SALAD /SALADS/ NATURAL OR PREPARED JUICES WITH FROZEN PULP?
(0) No (*go to q37*) (1) Yes

36.1 Food/drink	36.2 Quantity	36.3 Form of presentation
36.1.1		
36.1.2		
36.1.3		
36.1.4		
36.1.5		
36.1.6		
36.1.7		
36.1.8		

MESSAGES (*indicate in brackets the no. referring to the items of q 36.1*):
__________________________________( )
__________________________________( )
__________________________________( )
__________________________________( )
__________________________________( )
__________________________________( )
__________________________________( )
__________________________________( )
__________________________________( )
__________________________________( )

37. Is there any advertising in the establishment for INDUSTRIALIZED DRINKS?
(0) No (*go to q38*) (1) Yes

37.1 Food/drink	37.2 Quantity	37.3 Form of presentation
37.1.1		
37.1.2		
37.1.3		
37.1.4		
37.1.5		
37.1.6		
37.1.7		
37.1.8		

MESSAGES (*indicate in brackets the no. referring to item 37.1*):
__________________________________( )
__________________________________( )
__________________________________( )
__________________________________( )
__________________________________( )
__________________________________( )
__________________________________( )
__________________________________( )
__________________________________( )
__________________________________( )

38. Is there any advertising in the establishment for DESSERTS/ICE CREAMS?

(0) No *(Review filling in and note the time of finishing)*

(1) Yes

38.1 Food/drink	38.2 Quantity	38.3 Form of presentation
38.1.1		
38.1.2		
38.1.3		
38.1.4		
38.1.5		
38.1.6		
38.1.7		
38.1.8		

MESSAGES (*indicate in brackets the no. referring to q 38.1*):

_____________________________________( )
_____________________________________( )
_____________________________________( )
_____________________________________( )
_____________________________________( )
_____________________________________( )
_____________________________________( )
_____________________________________( )
_____________________________________( )

Review filling in and note the time of finishing

General comments:
